# Continuing education on child development in primary care: healthcare workers’ perspectives^*^

**DOI:** 10.1590/1980-220X-REEUSP-2023-0189en

**Published:** 2023-12-22

**Authors:** Rute Costa Régis de Sousa, Weslla Karla Albuquerque Silva de Paula, Fabia Alexandra Pottes Alves, Maria Ilk Nunes de Albuquerque, Grayce Alencar Albuquerque, Maria Wanderleya de Lavor Coriolano-Marinus

**Affiliations:** 1 Universidade Federal de Pernambuco, Programa de Pós-Graduação em Enfermagem, Recife, PE, Brazil.; 2 Universidade Federal de Pernambuco, Departamento de Enfermagem, Recife, PE, Brazil.; 3 Universidade Regional do Cariri, Programa de Pós-Graduação em Enfermagem, Crato, CE, Brazil.

**Keywords:** Child Development, Education, Continuing, Health Personnel, Primary Health Care, Health Promotion, Desenvolvimento Infantil, Educação Continuada, Pessoal de Saúde, Atenção Primária à Saúde, Promoção da Saúde, Desarrollo Infantil, Educación Continua, Personal de Salud, Atención Primaria de Salud, Promoción de la Salud

## Abstract

**Objective:**

To analyze the contributions of a continuing education with Primary Health Care professionals that promotes child development.

**Method:**

A continuing education intervention, utilizing a qualitative approach, was conducted among healthcare workers at a Primary Health Care facility in a low-income neighborhood in the city of Recife. The intervention consisted of eight workshops conducted between July and October 2019, with the participation of fifteen healthcare workers. All data from the focus groups were recorded, transcribed, and analyzed thematically using Bronfenbrenner’s bioecological model as the theoretical framework.

**Results:**

Through the continuing education intervention, healthcare professionals were able to reflect on their work processes and personal lives and propose actions to improve child development.

**Conclusion:**

The study findings highlight the significant impact of such interventions in changing perceptions and professional practices related to child development. Overall, this research provides valuable insights into the effectiveness of continuing education interventions for promoting healthy child development in primary care settings.

## RESUMO

**Objetivo:**

Analisar as contribuições de uma educação continuada com profissionais da Atenção Primária à Saúde que promova o desenvolvimento infantil.

**Método:**

Foi realizada intervenção de educação continuada, com abordagem qualitativa, entre trabalhadores de saúde de uma unidade de Atenção Primária à Saúde de um bairro de baixa renda da cidade do Recife. A intervenção consistiu em oito oficinas realizadas entre julho e outubro de 2019, com a participação de quinze profissionais de saúde. Todos os dados dos grupos focais foram gravados, transcritos e analisados tematicamente utilizando o modelo bioecológico de Bronfenbrenner como referencial teórico.

**Resultados:**

Por meio da intervenção de educação continuada, os profissionais de saúde puderam refletir sobre seus processos de trabalho e vida pessoal e propor ações para melhorar o desenvolvimento infantil.

**Conclusão:**

Os resultados do estudo destacam o impacto significativo de tais intervenções na mudança de percepções e práticas profissionais relacionadas ao desenvolvimento infantil. No geral, esta investigação fornece informações valiosas sobre a eficácia das intervenções de educação continuada para a promoção do desenvolvimento infantil saudável em ambientes de cuidados primários.

## RESUMEN

**Objetivo:**

Analizar los aportes de la educación continua con profesionales de la Atención Primaria de Salud que promueven el desarrollo infantil.

**Método:**

Se realizó una intervención de educación continua, con enfoque cualitativo, entre trabajadores de salud de una unidad de Atención Primaria a la Salud de un barrio de bajos ingresos de la ciudad de Recife. La intervención constó de ocho talleres realizados entre julio y octubre de 2019, con la participación de quince profesionales de la salud. Todos los datos de los grupos focales fueron registrados, transcritos y analizados temáticamente utilizando el modelo bioecológico de Bronfenbrenner como marco teórico.

**Resultados:**

A través de la intervención de educación continua, los profesionales de la salud pudieron reflexionar sobre sus procesos de vida laboral y personal y proponer acciones para mejorar el desarrollo infantil.

**Conclusión:**

Los resultados del estudio resaltan el impacto significativo de tales intervenciones en el cambio de percepciones y prácticas profesionales relacionadas con el desarrollo infantil. En general, esta investigación proporciona información valiosa sobre la efectividad de las intervenciones de educación continua para promover el desarrollo infantil saludable en entornos de atención primaria.

## INTRODUCTION

Child development is a multifaceted, continuous, and progressive process encompassing the acquisition of motor, cognitive, socio-emotional, linguistic, and various other skills^(1)^. Unfortunately, around 250 million children from low- and middle-income countries face the risk of not reaching their full developmental potential. This failure to achieve healthy development during childhood has far-reaching consequences in adulthood, impacting employability, productivity, mental health, and even criminality, thereby adversely affecting a country’s economic capacity^(2,3)^.

### Theoretical Framework

Bronfenbrenner’s Bioecological Theory of Human Development (BTHD) provides a comprehensive framework for understanding the developmental process, emphasizing the interaction of four key components: process, person, context, and time. These components collectively form the Process-Person-Context-Time (PPCT) model, with their interplay shaping developmental outcomes^(4)^.

The Person component recognizes that individual attributes contribute to a child’s overall developmental trajectory. The Process component highlights the importance of proximal processes, such as reciprocal interactions between a child and their surroundings. The Context component emphasizes the various contexts in which development occurs. The Time component acknowledges the role of historical and biological time.

Bronfenbrenner’s theory and the PPCT model provide a holistic framework that considers the individuals’ complex interplay, their interactions with the environment, and the larger socio-cultural and historical context in shaping human development. By understanding and analyzing these components, researchers, practitioners, and policymakers can gain valuable insights into how various factors influence and impact individuals’ developmental outcomes.

### Healthcare Workers and Child Development Promotion

There is a substantial body of evidence supporting the positive impact of interventions aimed at promoting child development^(5,6)^. However, the majority of these interventions primarily target mothers, caregivers, or families in general, with limited focus on healthcare workers’ perspectives. Research conducted across continents, including North and South America, Europe, Africa, and Australia, indicates that healthcare workers from various professions face challenges in implementing actions that support child development in their practice^(7,8,9,10,11)^.

Nevertheless, there is encouraging evidence suggesting that professional education on child development can yield positive outcomes^(12,13)^. Studies have shown that interventions focusing on enhancing healthcare workers’ knowledge and skills in this domain can be effective in promoting optimal child development outcomes.

It is important to recognize that while interventions targeted at mothers, caregivers, and families play a vital role in supporting child development, involving healthcare workers is crucial for comprehensive and integrated care. Healthcare workers are in a unique position to engage with families and provide guidance and support during crucial stages of child development. By equipping healthcare workers with the necessary knowledge and tools, we can enhance their ability to identify and address developmental needs effectively.

Therefore, this study aims to analyze the contributions of a continuing education intervention targeting Primary Health Care professionals that promotes child development. Furthermore, it seeks to demonstrate the practical application of the PPCT model proposed by BTHD.

## METHOD

### Study Design

In this study, a qualitative approach is employed to conduct an intervention study, guided by the PPCT model theoretical framework proposed by BTHD^(4)^. This article followed the guidelines recommendations proposed by the COnsolidated criteria for REporting Qualitative research (COREQ).

#### Eligibility Criteria, Population, and Sample

All healthcare workers responsible for providing care to children aged 0 to 6 years old at a Primary Health Care service in a low-income neighborhood in Recife municipality were eligible to take part in the intervention. Out of a total of twenty-nine workers employed at the primary care service, eighteen healthcare workers were eligible and invited to take part in the intervention. Only three of them declined to participate in the study. Therefore, the final sample was selected for convenience and consisted of fifteen healthcare professionals who accepted to participate in the intervention, which comprised participation in workshop group meetings and the implementation of proposed activities with health users in the workplace. There were two registered nurses, one dietician, one occupational therapist, one speech therapist, one nurse technician, seven community health workers, and one oral health assistant.

### Local

The study was conducted between July and October 2019 at the community center situated adjacent to the Primary Health Care service. This particular location was selected by healthcare workers due to its proximity to the Primary Health Care service and its spacious area, which provided the necessary privacy for holding meetings.

### Intervention and Data Collection

All healthcare workers at the aforementioned primary care facility had prior familiarity with one of the authors. This familiarity arose because the facility serves as a practical training site for nursing students at the *Universidade Federal de Pernambuco* (UFPEv), and one of the authors holds a position as a professor and nursing practice supervisor at UFPE.

During a scheduled meeting between one of the authors and Primary Health Care workers, held as part of the university’s efforts to enhance teaching and service integration, workers identified a specific need for more extensive knowledge and understanding of child development. As a result, the continuing education intervention described here was initiated in response to the expressed demand from healthcare professionals at the Primary Health Care facility.

The intervention consisted of eight workshops, and each workshop addressed a different topic related to child development. The topics of each of the eight workshops were in this order: 1^st^ – child development: awareness and initial concepts; 2^nd^ – socio-emotional development; 3^rd^ – Child Health Handbook; 4^th^ – Positive parenting practices; 5^th^ – Education and communication in health: qualified listening and empathy; 6^th^ – Action plan development; 7^th^ – Presentation of experiences; and 8^th^ – Intervention assessment. Each workshop had an average duration of three hours and was conducted with fifteen-day interval between them.

A different research team member was responsible for conducting each group meeting, and two of the authors participated in all groups. All research team members had prior knowledge in qualitative research, specifically with the focus group technique. The research team was composed by nursing professors and graduate nursing students. With the exception of the first and last workshops, each session was divided into five distinct segments: discussion of the previous meeting’s activity; topic presentation; topic critical analysis; coffee break; and an introduction to the new activity.

Each activity proposed during the workshops was directly related to the corresponding workshop topic and was conducted collaboratively among professionals, actively involving health service users. Various educational methodologies, such as videos, puzzles, gamification, and role-play, were employed to present and analyze the topics. The entire duration of each meeting was audio recorded, with the exception of the coffee break and periods of non-discussion when the recording was paused. The recording time lasted approximately eighteen hours in total and about two hours for each workshop.

### Data Analysis and Treatment

All recorded audios were transcribed verbatim by a research team member. The transcripts were meticulously reviewed in conjunction with the audio recordings by one of the authors who simultaneously read the transcripts and listened to the corresponding audio. Thematic analysis^(14)^ was utilized to analyze the data. Two of the authors collaborated in reading and coding each transcript line by line. The codes were then subjected to discussions, refinements, and grouping based on their similarities. Finally, they were assigned to topics and subtopics using the PPCT model as a theoretical framework. Due to the COVID-19 pandemic (2020–2021), which led to the suspension of group meetings, it was not possible to conduct a group feedback session regarding the results.

### Ethical Aspects

This research adhered to the ethical guidelines outlined in Resolution 466/12. Ethical approval was obtained on 2020, under approval number 4.324.274. Prior to the first workshop, all participants were thoroughly informed about their right to withdraw, anonymity guarantee, and the purposes, procedures, risks, and benefits of the research. They provided their informed written consent, indicating their willingness to participate. To ensure participant anonymity, each healthcare professional participating in the study was assigned a unique identification number. In-text citations within the research paper utilize the acronym ‘HP’ (Healthcare professional) followed by the corresponding assigned number.

## RESULTS

A total of fifteen healthcare workers participated in the intervention, including two registered nurses, one dietician, one occupational therapist, one speech therapist, one nurse technician, seven community health workers, and one oral health assistant. All participants identified as female, accounting for 100% of the sample. Most participants (75.86%) were mothers themselves, and nearly half (48.27%) held a college degree. Participants’ mean age was 42.38 years (SD = 9.08), and their experience in Primary Health Care ranged from two to 24 years.

### Previous Knowledge and Healthcare Professionals’ Practice

Healthcare professionals shared their knowledge and practices regarding child development, with a specific focus on the perspectives of the primary actors responsible: mothers and grandmothers. This characteristic, referred to as the Person component by the PPCT model, serves a dual purpose, as it not only identifies individuals’ biological sex but also encompasses the social context. Social perceptions of female and male figures differ, with females often being closely associated with the caregiving role.

Health professionals perceived the mother’s young age and the grandmother’s involvement in child care as negative factors that may pose risks for both the child and the grandmother.

*In the community, we see an adolescent of twelve years having children. (...) adolescents. A child taking care of another child.* (HP 1)

*I think it’s very risky to delegate the care of a child to a grandmother. (...) and then, you hand over the responsibility of a baby being raised in a situation like this. It’s risky for the child and the grandmother. (...) it compromises child development.* (HP 3)

Health professionals perceived their participation as having a co-responsible role in promoting child development negatively. They did not view themselves as agents of change and expressed feelings of weariness, hopelessness, and a sense of powerlessness regarding the child development process.

*And we, as healthcare professionals who are on the forefront, (...) who are even more in contact with the community, feel this tiredness, this weariness.* (HP 3)

Concerning child characteristics, which falls under the Person component of the PPCT model, healthcare professionals emphasized the challenges faced by children with attention deficit hyperactivity disorder (ADHD) and child aggressiveness.

*And she needed a lot of patience because he was kind of hyperactive.* (HP 1)

*(...) there was one who said that she had a lot of difficulties controlling her son because he is very aggressive with other children, he plays, plays, plays and then lashes out at everyone.* (HP 2)

Professionals discussed the perceptions regarding the relationships between caregivers and children, representing the Process component. They highlighted aspects such as caregiver neglect and insufficient resources to meet children’s physical and emotional needs. Through their comments, caregivers were portrayed as being at fault and responsible for the negative issues affecting child development. Consequently, the interaction between healthcare professionals and caregivers appeared to be characterized by judgment regarding parental responsibility and behavior, rather than fostering a supportive relationship, namely a positive process between caregivers and healthcare workers.

*The child is either taken care of by the grandmother or an older sibling. At the end of the day, this child will not be cared for by anyone.* (HP 4)

Another process that emerged from healthcare professionals’ comments was the interaction between children and technology. According to professionals’ perceptions, caregivers held the belief that using technology has a positive impact on child development. In some cases, caregivers even used technological devices to divert their attention from the child, neglecting their active involvement in child development.

*And mothers think that children are being stimulated by watching videos on YouTube.* (HP 2)

Participants highlighted the influence of a low socioeconomic status on the relationship between caregivers and children, which correspond to the Context component of the PPCT model. They emphasized that the economic circumstances directly impact the caregiving dynamics and interactions between caregivers and children.

*These children (...) they are not treated well. The stress of not having enough to eat, not having anything to give, not being able to provide for will generate emotional stress in the father and mother that, consequently, will be taken out of the child.* (HP 3)

Another contextual factor that emerged from participants’ comments and has an impact on child development is the lack of available daycare centers. This limitation forces caregivers to seek alternative arrangements that may not always be ideal for children, as their primary focus is to be able to work while ensuring some level of care for their children.

*And it is not a qualified education. It is a person from the community who is willing, (...) who is willing to look after the child at that time so that the mother can work, right? But without the slightest qualification.* (HP 5)

### Reflections About New Knowledge and Skills in Professional Practice and Personal Life

Participants highlighted the potential for overcoming challenges and obstacles through community engagement, particularly when influential individuals in strategic positions are actively involved and sensitized on this issue.

*Then, from the moment I manage to convince the person who is most involved, then it becomes easier.* (HP 1)

Another reflection on professional practice emphasized the significance of effective communication with patients, characterized by qualified listening, non-judgmental attitudes, and respect for their personal choices. It recognized that there may be differences between healthcare professionals’ and users’ perspectives, highlighting the need for professionals to be prepared to listen and welcome users without interrupting or criticizing them.

*It’s also good to include the issue of our point of view there so as not to interfere. Because then, what we experience, as a person, is one thing, and what we experience as a professional is another.* (HP 7)

Participants also engaged in reflection on the distinction between working with children and adults, noting that working with children was often perceived as easier due to their openness and lack of solidified opinions on various subjects. In contrast, working with adults was considered more challenging due to preconceived concepts and beliefs that could hinder change. However, participants acknowledged that although children may be more receptive to new knowledge, they lacked the power to drive significant changes in family lifestyles. Therefore, it was emphasized that it is crucial to work with all family members to ensure the effective implementation of desired changes.

*So, I think working with the family is better, because in relation to healthy eating, (...) but when we do it just with the kid, then when recess time comes there is the school lunch, but when they open their lunch bag, they take out a bottle of soda and chips. So, you feel helpless, right? When you bring the family in to talk together, showing why a healthy diet, I believe the result is better.* (HP 12)

### Reflections on Personal Experiences

The professionals also engaged in personal reflections on their own childhood experiences, drawing upon these reflections to inform their understanding and appreciation of children’s emotions. By considering their own experiences, the professionals recognized the significance of acknowledging and valuing the feelings of children.

*(...) I would have liked to have had this upbringing, for me it is the ideal way, (...) because for a child it is not easy for them to forget something they want very much, no matter how much the adult explains it, but for children, it is not easy, (...)*. (HP 9)

Participants further engaged in reflection on the significance of striking a balance between overprotection and fostering the development of autonomy in children, recognizing the importance of this balance for promoting healthy development.

*(...) I think I was crazy, and I prepared myself so much to be a mother that I unloaded so much of myself. And then today I see that there is something about development that, like, I put it in a box, and today it has repercussions.* (HP 13)

### Changes to Professional Practice

In the final workshop, professionals were encouraged to reflect on the changes they had observed in their own professional practice and to propose actions aimed at promoting child development within their community. These actions were derived from the knowledge and practices those professionals had acquired throughout the educational process. This reflective exercise enabled professionals to consolidate their learning and translate it into tangible initiatives that would contribute to child well-being and development in their local community.

As part of the proposed changes by healthcare workers, it was suggested to develop a video on child development that could be used during health education activities with the community. The primary objective of this video would be to provide individuals and families with valuable information and guidance on various aspects of child development. Moreover, professionals proposed creating a customized script to be used during home visits, which would be tailored according to the age group of the children in each household.

*(...) a video including all of the information of, at most, about 6 minutes. Because then they, the CHW (community health worker) or the person who was going to make the presentation play the video and complement it with questions, you know, they could have an exchange, right?* (HP 10)

Participants also put forward a suggestion to create a play kit consisting of homemade toys. This play kit would serve two purposes: first, it would assist in assessing child development; and second, it would serve as an incentive for parents and caregivers to actively engage in play with children using the resources available at home.

*(...) a small kit like that, which you could take to people’s houses (...) that had toys, you know, that you could (...) assess child development for the mother to observe (...)* (HP 1)

Among the changes perceived by participants, increased motivation was another notable outcome resulting from the educational strategy. Professionals expressed a heightened sense of enthusiasm and excitement in promoting changes in their professional practices and applying the knowledge and skills acquired throughout the educational process.

*(...) because when we take a course, we always come out with this feeling [...] of having a small itch that is there messing with us (...)* (HP 8)

## DISCUSSION

This study aimed to analyze the contributions of a continuing education intervention with Primary Health Care workers to child development promotion and explore how the findings align with BTHD and its PPCT model. The professionals involved in the study acknowledged that the educational intervention built upon their existing knowledge and practical experiences enabled them to acquire new knowledge and skills that were relevant and applicable to the local context. They recognized the value of the knowledge gained during each session and actively explored ways to transfer and apply it within their work settings through collaborative actions. The study findings highlight the importance of contextually grounded education and collaborative approaches in promoting effective knowledge translation and professional development within Primary Health Care settings.

Professionals’ comments reflected a consistent perception regarding the role of women in caregiving, with specific attention given to mothers’ age. Adolescent mothers were generally perceived as less capable of effective parenting compared to those who became pregnant in adulthood. This perception corresponds with existing research that has demonstrated negative impacts on child developmental outcomes of adolescent mothers^(15)^. The lower maternal self-efficacy reported among adolescent mothers further supports this perception. This alignment with the PPCT model underscores the influence of individual characteristics, specifically the mother’ age, on child developmental outcomes.

Professionals’ recognition of the significant role played by grandmothers in child developmental outcomes aligns with the literature. The presence of grandmothers in the care network has been shown to positively impact child development. A study conducted in Pakistan specifically highlighted the positive effects of close grandmother involvement in the care of children at 12 months of age. This involvement was associated with better cognitive performance, fine motor skills, and emotional development in children^(16)^.

Another study in China pointed out that the grandmother’s presence as a caring figure for children was associated with developing a better bond between mother and baby. However, positive outcomes only occurred when the grandmother showed neutral behavior, mainly involving observation and support when requested^(17)^. This leads to the reflection that the child’s benefits from the care relationship with the grandmother will also depend on the mother and daughter relationship.

Healthcare professionals had difficulties pointing out positive proximal processes between children and their main caregivers: mother and grandmother. The main interactions would occur between the child and technology. This situation was also found in a prior study investigating parenting practices in rural regions of China^(18)^.

The absence of positive proximal processes may result in delays in neurological, language, social, and emotional development^(19)^, and may also have long-term repercussions in adult life with low educational and employability levels, thus negatively impacting a country’s economy^(3)^. Several factors explain this reality, such as low socioeconomic conditions, which induce stress in caregivers, and individual factor, such as maternal mental health and educational level^(20)^.

A further factor concerns personal and cultural beliefs about how interactions with children should occur. In a study that investigated parental beliefs and attitudes about the need to play with children, parents cited play based on physical, electronic toys, or digital media as good for child development and education. The authors concluded that interactive parent-child play promotes development and school readiness skills^(21)^.

Knowledge and attitudes can be changed through interventions to modify inappropriate beliefs. Several interventions aimed at parenting practices, such as proximal processes, are effective in modifying developmental outcomes such as language, school readiness, and cognitive and socio-emotional skills^(22)^.

A context addressed in professionals’ discourse is the absence of daycare centers in the community. According to healthcare professionals’ view, children would be more encouraged in daycare centers than at home and would be receiving qualified care. This perception is in line with what the literature shows that the care offered in daycare centers has a positive impact on the outcome of development, as it cancels out inequalities in development associated with caregiver individual characteristics such as low maternal education or the context, such as community violence or low socioeconomic status^(23)^.

Some professionals highlighted contextual factors as the most relevant for the development outcome. This perception goes against the BTHD, which sets the proximal processes as the most powerful component that influences development, being able to reduce or increase the negative impact of the context.

When analyzing the intervention contributions, we can see that if at first healthcare professionals were unmotivated and could not perceive themselves as agents of change capable of positively influencing the outcome of development, throughout the educational process, they reflected on the importance of engaging the community, especially key persons (the most influential figures in the community respected by others as leaders). Because they have greater influence with users, these people can help build bridges between the community and the health service and become knowledge multipliers, an initiative that has already been used successfully^(24)^.

In addition to the importance of attracting community leaders to health promotion, healthcare professionals also reflected on the need to involve the whole family in child development promotion, making it a complex intervention, as the family is composed of individuals of different ages and sexes, which demand different educational approaches. The engagement of all family members will result in a better reach and success of interventions^(25)^.

Another reflection made on professional practice was on communication with users, i.e., the proximal processes between caregivers and healthcare professionals. Healthcare professionals perceived the need to change the way they communicate with caregivers, to develop a listening attitude without making judgments, without interrupting, without criticizing and always respecting users’ and caregivers’ opinions and beliefs^(26)^.

Creating a home visit script aimed at promoting child development was suggested as one of the tools that could be used and adapted according to children’ age. Home visit programs for pregnant women and mothers are a tool to promote child development that has been widely used, and their effectiveness has been described in the literature^(27)^.

In the Primary Health Care unit where the intervention was carried out, there was not a specific program of home visits whose objective was to promote child development. Home visits are featured as a routine part of the work process. Therefore, using a script that contains the appropriate developmental milestones for each age and a summary of how to assess each milestone, amongst others, would allow professionals to know the main points to be addressed regarding each age group, optimizing time and enabling a standardization between home visits in all family households^(28)^.

In addition to the script, another suggested resource was a play kit with toys to analyze the developmental milestones and encourage caregivers and parents to play with their children. These materials could be built from materials present in caregivers’ daily lives. The literature shows that plays and toys appropriate for children’s age promote the acquisition of socio-emotional, cognitive, language, and self-regulation skills. The act of playing is also related to an increase in the production of neurotransmitters that neutralize the effect that toxic stress produces on the body. Thus, playing becomes even more important in children living in vulnerable situations, such as those assisted by the participants of this study^(29)^.

Using toys that caregivers could build is important since the environment where professionals develop their practice is highly vulnerable to social problems. Using homemade toys does not diminish the benefits that the act of playing brings. On the contrary, recent studies indicate that electronic toys allow fewer moments of interaction between children and caregivers. Moreover, the lower availability of toys would result in longer periods of play with a single toy, allowing for improved focus and creativity^(30)^.

One limitation of this study is the absence of feedback from participants regarding the final results. This limitation arose due to the constraints imposed by the COVID-19 pandemic, which also hindered the monitoring of the implementation of actions proposed by healthcare professionals and the assessment of their impact on families and caregivers.

## CONCLUSION

The continuing education intervention facilitated critical reflections among healthcare professionals regarding their work processes and personal lives. As a result of the intervention, healthcare professionals experienced changes in their professional practices and proposed various actions to promote child development. The intervention also led to increased motivation among participants, fostering optimism about potential changes within their community.

Healthcare professionals’ discourses encompassed all components of the PPCT model, but the Time component was mentioned less frequently. Overall, the study findings demonstrate how the educational intervention aligned with the PPCT model, enabling professionals to reflect on their practices, propose effective actions, and experience positive changes in their professional roles.

This research findings underscore the importance of continuing education interventions in healthcare settings to promote early child development. The positive impact on healthcare professionals’ critical thinking and practice changes suggests that similar interventions should be encouraged and supported in the Primary Health Care service. Healthcare professionals can leverage the insights gained from this research to implement community-based initiatives that prioritize child development, ultimately leading to improved child development outcomes. It also highlights the potential role of existing local policies such as the health family strategy as an appropriate environment for interventions aimed at comprehensive child development promotion.
